# An endothelial-related prognostic index for bladder cancer patients

**DOI:** 10.1007/s12672-024-00992-4

**Published:** 2024-04-25

**Authors:** Deng-xiong Li, Rui-cheng Wu, Jie Wang, Qing-xin Yu, Zhou-ting Tuo, Lu-xia Ye, De-chao Feng, Shi Deng

**Affiliations:** 1 Department of Urology, Institute of Urology, West China Hospital, Sichuan University, Chengdu, 610041 Sichuan China; 2 Department of Pathology, Ningbo Clinical Pathology Diagnosis Center, Ningbo, China; 3 Department of Urology, The Second Affiliated Hospital of Anhui Medical University, Hefei, 230601 China; 4 Department of Public Research Platform, Taizhou Hospital of Zhejiang Province Affiliated to Wenzhou Medical University, Linhai, China

**Keywords:** Endothelial cells, Bladder cancer, Single-cell, Precision medicine

## Abstract

**Background:**

Within the tumor microenvironment, endothelial cells hold substantial sway over bladder cancer (BC) prognosis. Herein, we aim to elucidate the impact of endothelial cells on BC patient outcomes by employing an integration of single-cell and bulk RNA sequencing data.

**Methods:**

All data utilized in this study were procured from online databases. R version 3.6.3 and relevant packages were harnessed for the development and validation of an endothelial-associated prognostic index (EPI).

**Results:**

EPI was formulated, incorporating six genes (CYTL1, FAM43A, GSN, HSPG2, RBP7, and SLC2A3). EPI demonstrated significant prognostic value in both The Cancer Genome Atlas (TCGA) and externally validated dataset. Functional results revealed a profound association between EPI and endothelial cell functionality, as well as immune-related processes. Our findings suggest that patients with low-risk EPI scores are more likely to respond positively to immunotherapy, as indicated by immune checkpoint activity, immune infiltration, tumor mutational burden, stemness index, TIDE, and IMvigor210 analyses. Conversely, individuals with high-risk EPI scores exhibited heightened sensitivity to cisplatin, docetaxel, and gemcitabine treatment regimens.

**Conclusion:**

We have effectively discerned pivotal genes from the endothelial cell perspective and constructed an EPI for BC patients, thereby offering promising prospects for precision medicine.

**Supplementary Information:**

The online version contains supplementary material available at 10.1007/s12672-024-00992-4.

## Introduction

Bladder cancer (BC) stands as the 6th most prevalent malignancy and ranks 9th among causes of cancer-related mortality in men [1, 2]. Despite the availability of diverse therapeutic modalities, the mortality rate among BC patients remains elevated, particularly in developing nations [3]. Consequently, a significant proportion of patients do not experience enhanced prognoses, bearing substantial physical, emotional, and financial burdens [4]. For instance, radical cystectomy, a common treatment approach, is associated with considerable harm and a marked deterioration in the quality of life, yet the post-surgical survival outcomes remain unfavorable [5]. Thus, the current advancements are far from meeting the desired standards. To address this quandary, researchers are diligently investigating the underlying mechanisms of BC with the aim of preventing its onset [6]. Among these aspects, the tumor microenvironment has emerged as a focal point of considerable research interest [7, 8]. Leveraging single-cell analysis techniques, we can dissect the precise functions of individual cell types, thereby facilitating the discovery of novel therapeutic approaches [9, 10]. Beyond uncovering new mechanistic insights and therapeutic targets, the judicious utilization of existing treatment modalities has become a paramount concern for clinicians [11]. Addressing this challenge necessitates the utilization of robust markers, including histological features and the tumor microenvironment, to guide the personalized selection of optimal therapeutic strategies for individual patients [5, 12]. Simultaneously, the identification of clinical and molecular factors (such as genetic profiles and clinical stage) bearing significant prognostic significance has empowered healthcare practitioners in making informed clinical decisions [13, 14].

An organized microvasculature comprises an endothelial cell layer, underpinned by a basement membrane and ensconced by perivascular cells, typically pericytes [15]. This microvascular framework serves to nourish the urothelium and the underlying lamina propria and muscularis propria [16, 17]. However, within the context of tumorous tissue, endothelial cells are often surrounded by a diminished population of pericytes, resulting in compromised inter-endothelial cell junctions, hence, increased vascular permeability [18]. In urological malignancies, a signature based on endothelial cell characteristics holds potential for predicting the prognosis and therapeutic outcomes of individuals diagnosed with kidney clear cell cancer [19]. The hypoxic and nutrient-deprived milieu induced by the leaky tumor vasculature amplifies the invasive attributes of bladder cancer cells [20]. Furthermore, differential drug penetration from blood vessels results in variations in drug concentrations within the BC tissue, with BC cells located closer to the vasculature being exposed to higher drug concentrations compared to those situated further away [21]. Concurrently, chemotherapy-induced cell death often leads to the emergence of a sizable population of chemotherapy-resistant cancer stem cells within the residual BC tissue [22, 23]. Consequently, the presence of endothelial cells within the tumor microenvironment exerts a profound impact on both the prognosis and treatment responses in BC.

To address these pivotal aspects, we systematically collected and analyzed BC data obtained from online databases. Our objective was to discern key genes, from the vantage point of endothelial cells, and subsequently construct an endothelial-centric prognostic index. Subsequently, a variety of methodologies were deployed to rigorously validate both the index and the identified key genes.

## Materials and methods

### Endothelial-related genes validation

To identify endothelial-related genes, data for the analysis encompassing BC and normal tissue samples were sourced from The Cancer Genome Atlas (TCGA) database (www.gdc.cancer.gov, TCGA). Differentially expressed genes were identified based on rigorous criteria: a P-value < 0.05 and an absolute log2-fold change exceeding 1. This analysis was conducted utilizing the ‘limma’ package and encompassed 19 normal tissue samples and 414 BC samples, as previous description [24]. Furthermore, stringent patient inclusion criteria were enforced, wherein individuals with postoperative survival durations less than 30 days, those with non-transitional cell cancer subtypes, or those lacking survival outcome data were excluded from the study. Then, the determination of endothelial cell content for each included BC sample within the TCGA dataset was accomplished through the utilization of the xCELL platform (https://xcell.ucsf.edu/) [25]. A stringent criterion, based on the Pearson correlation analysis, was employed, necessitating that endothelial-related genes exhibit |coefficients|> 0.3 and a P-value < 0.05. Subsequently, we accessed endothelial cell markers from the Tumor Immunotherapy Gene Expression Resource (TIGER) database (http://tiger.canceromics.org), selecting markers with |log2FoldChange|> 0.3, derived from single-cell data analysis reported in publicly available publications. Notably, a prior study made the original data on endothelial-related markers in BC publicly available [26]. Ultimately, we intersected the three sets of differential genes to ensure the selected genes are related to tumor endothelial cells.

### Construction and validation the endothelial-related prognostic index

All differentially expressed genes were integrated into the lasso regression model, subsequently leading to the construction of the endothelial-related prognostic index (EPI), featuring six genes (CYTL1, FAM43A, GSN, HSPG2, RBP7, and SLC2A3). The prognostic utility of the EPI was assessed through Kaplan–Meier survival analysis within both internal and external datasets, as well as within distinct clinical subgroups. Concurrently, factors exhibiting a P-value < 0.1 in the univariable Cox regression model were included in the multivariable Cox regression model to ascertain the EPI's independent prognostic significance. Additionally, the protein–protein interactions of these six genes were investigated using the GeneMANIA platform (www.genemania.org) [27]. In order to illustrate the discriminatory capacity of the EPI, we generated risk score plots contingent upon the risk score and survival outcomes, likes previous work [28]. Furthermore, we obtained external validation datasets, namely GSE32894, from the Gene Expression Omnibus (GEO) database (https://www.ncbi.nlm.nih.gov), augmenting the robustness of our analyses. GSE32894 contained gene expression information and follow-up results for 224 bladder cancer samples and most of samples diagnosed at an early clinical stage [29].

### Function and immune-related analysis

Leveraging the TCGA dataset in conjunction with the EPI, we conducted comprehensive Gene Ontology (GO) enrichment analysis encompassing molecular function (MF), biological process (BP), and cellular component (CC). The stringent criteria for GO term selection encompassed a P-value < 0.05 and a Q-value < 0.05. Furthermore, the enriched pathways from the Kyoto Encyclopedia of Genes and Genomes (KEGG) were identified adhering to identical significance thresholds. Additionally, REACTOME pathways were meticulously curated via Gene Set Enrichment Analysis (GSEA), with significance thresholds set at P-value < 0.05 and FDR < 25%.

In the TCGA dataset, we conducted a comparative analysis of 34 immune checkpoints between the high- and low-risk score groups. Moreover, guided by the xCELL results, we assessed the discrepancies in infiltrated immune cell proportions between these risk score groups. Additionally, we computed the tumor mutational burden (TMB) using the ‘maftools’ package, subsequently contrasting the TMB scores between the high- and low-risk score groups. Furthermore, we evaluated the mRNA expression-based stemness index (mRNAsi) score, comparing it between these two risk score categories [30]. To prognosticate responses to immunotherapy, we harnessed the TCGA-BC cohort and leveraged the Tumor Immune Dysfunction and Exclusion (TIDE) algorithm [31]. This facilitated predictions and inter-group comparisons of immunotherapy responses. Subsequently, we drew upon data from the IMvigor210 trial (EGAD00001003977), encompassing patients with advanced or metastatic BC treated with an anti-PD-L1 agent (atezolizumab), to gauge the association between therapeutic outcomes and the EPI [32]. Furthermore, we explored the predictive utility of EPI in chemotherapy response. The half-maximal inhibitory concentration (IC50), calculated using the “pRRophetic” package within the R software, served as the primary endpoint for assessing chemotherapy responses and drug sensitivity [33]. The data source for the “pRRophetic” algorithm was gene expression levels from each TCGA sample. These IC50 values were also compared between the EPI high- and low-risk score groups.

### Statistical analysis

Statistical analyses of continuous variables involving three or more groups were conducted using either one-way ANOVA or the Mann–Whitney U test, contingent upon data normality and variance quality. When comparing quantitative data between two groups, the Student’s t-test was employed. All presented data are expressed as mean ± standard deviation (SD). A significance threshold of P < 0.05 was applied to all analyses, which were executed using R version 3.6.3 and relevant packages. ns, P ≥ 0.05; *, P < 0.05; **, P < 0.01; ***, P < 0.001.

## Results

### Construction the index and basic data

**Fig. 1:**
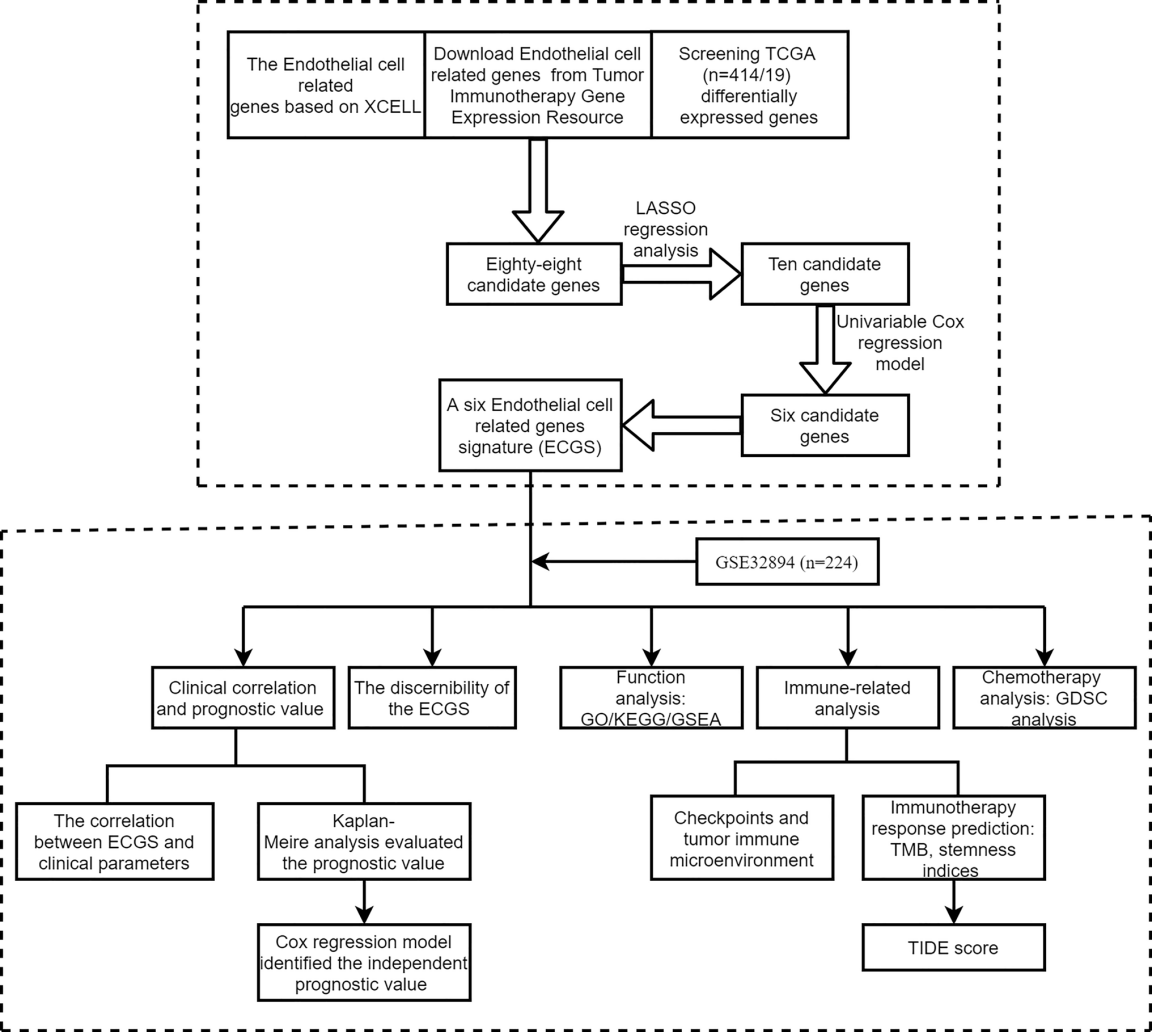
The workflow of this study

**Fig. 2:**
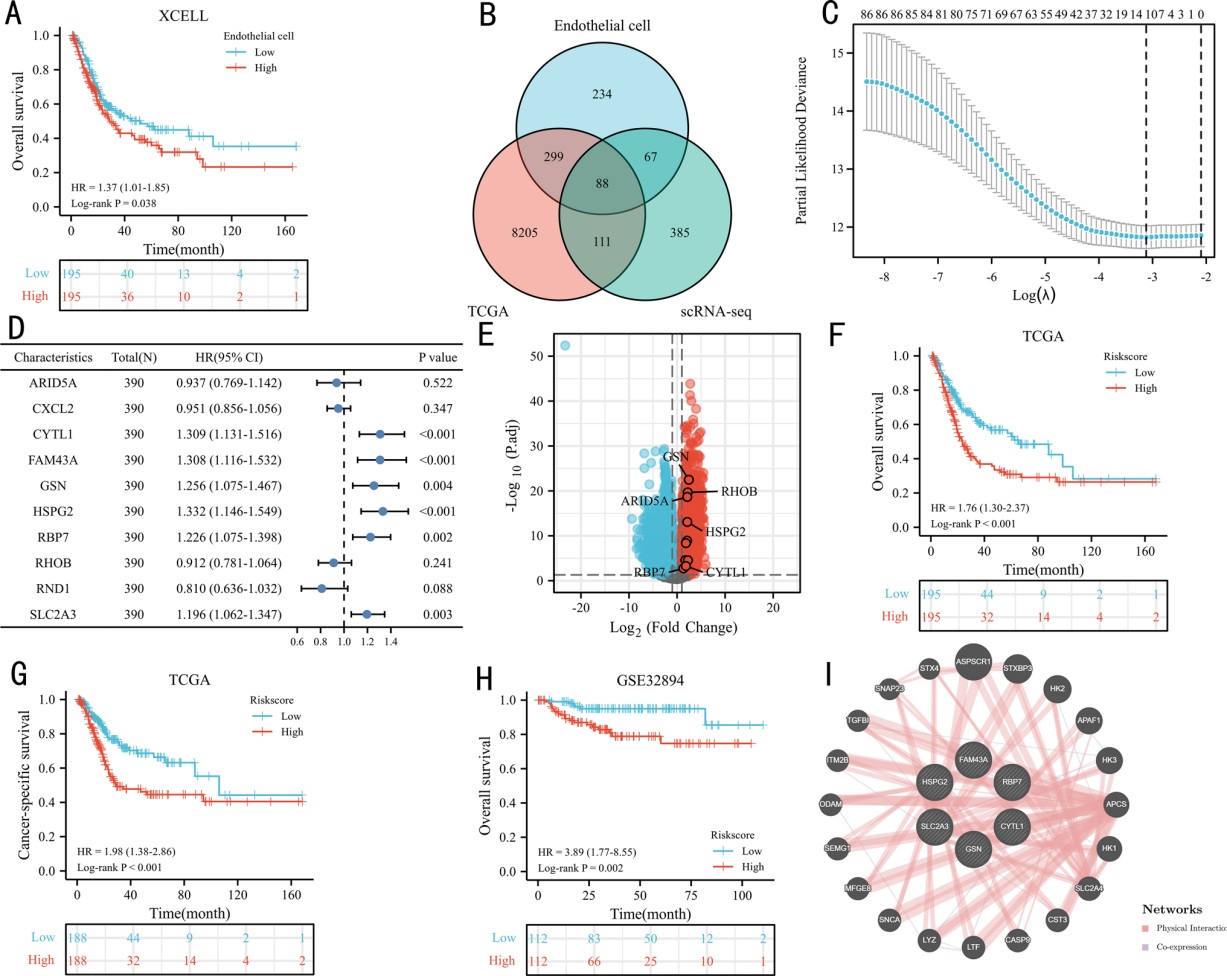
The prognostic value of endothelial cells in TCGA dataset (**A**), the co-expressed genes (**B**), the cross-validation to determine the optimal penalty parameter lambda (**C**), the prognostic value of these ten genes in overall survival (OS) according to the results of univariable Cox regression analysis in TCGA dataset (**D**), volcano plot of expression levels of these six genes (**E**), the Kaplan‒Meier analysis results of OS in TCGA dataset (**F**), the Kaplan‒Meier analysis results of cancer-specific survival in TCGA dataset (**G**), the Kaplan‒Meier analysis results of OS in GSE32894 dataset (**H**), the protein–protein interaction network (**I**)

Figure 1 showed the workflow of our study. As shown in Fig. 2A, patients with high endothelial cell infiltration had significantly worse overall survival (OS) than those with low endothelial cell infiltration (P = 0.038). There were 651 endothelial markers extracted from TIGER (Supplementary Table 1). The results of Pearson correlation analysis identified 689 endothelial-related genes (Supplementary Table 2). According to the results of VennDiagram, there were 88 differentially co-expressed endothelial-related genes screened (Fig. 2B). Figure 2C exhibited the results of lasso regression analysis, which selected 10 genes finally. Then, six of these genes presented significant prognostic value in TCGA dataset based on the results of univariable Cox regression model (Fig. 2D). All these six genes were high expressed in BC tissues than the counterpart normal samples (Fig. 2E). The EPI risk score = CYTL1*0.0764 + FAM43A*0.1213 + GSN*0.0209 + HSPG2*0.1908 + RBP7*0.1087 + SLC2A3*0.0042–1.2302. According to the median value of the risk score, we divided patients in TCGA and GSE32894 into high- and low-risk score groups, respectively. In validation analysis, the EPI could predict the OS (Fig. 2F, P < 0.001) and cancer-specific survival (Fig. 2G, P < 0.001) of BC patients in TCGA dataset. This outcome also identified in GSE32894 (Fig. 2H, P = 0.002) datasets. Figure 2I presented the interacting proteins of EPI, including ASPSCR1, STXBP3, HK2, and so on.

After excluding unqualified samples, a total of 390 patients in TCGA dataset included in this study. In TCGA dataset, there were significant differences in World Health Organization (WHO) grade, American Joint Committee on cancer (AJCC) stage, lymph node metastasis, T stage, OS, and cancer-specific survival between high- and low-risk score groups (Table 1). The detail information of GSE32894 datasets was showed in Supplementary Table 3.

**Table 1:** The clinicopathological characteristics of the TCGA included patients

Characteristic	Low risk-score	High risk-score	p
n	195	195	
Age, mean ± SD	67.3 ± 10.9	68.49 ± 10.18	0.268
BMI, mean ± SD	26.54 ± 5.6	27.28 ± 5.52	0.224
Smoking history, n (%)			0.924
No	52 (13.8%)	53 (14.1%)	
Yes	138 (36.6%)	134 (35.5%)	
Sex, n (%)			0.300
Female	46 (11.8%)	56 (14.4%)	
Male	149 (38.2%)	139 (35.6%)	
WHO grade, n (%)			0.008
High grade	178 (46%)	191 (49.4%)	
Low grade	15 (3.9%)	3 (0.8%)	
AJCC stage, n (%)			< 0.001
AJCC stage I–II	86 (22.2%)	37 (9.5%)	
AJCC stage III–IV	107 (27.6%)	158 (40.7%)	
Lymph node metastasis, n (%)			0.018
N+	49 (14%)	75 (21.4%)	
N0	121 (34.5%)	106 (30.2%)	
Distant metastasis, n (%)			0.052
M0	115 (59%)	70 (35.9%)	
M1	3 (1.5%)	7 (3.6%)	
T stage, n (%)			< 0.001
T3_4	96 (26.7%)	149 (41.5%)	
Ta_2	74 (20.6%)	40 (11.1%)	
Overall survival, n (%)			< 0.001
Alive	127 (32.6%)	92 (23.6%)	
Dead	68 (17.4%)	103 (26.4%)	
Cancer-specific survival, n (%)			0.001
Alive	147 (39.1%)	113 (30.1%)	
Dead	44 (11.7%)	72 (19.1%)	

*AJCC* American Joint Committee on cancer, *BMI* body mass index, *SD* Standard deviation, *WHO* World Health Organization, *n* Number

### Validation the index

**Fig. 3:**
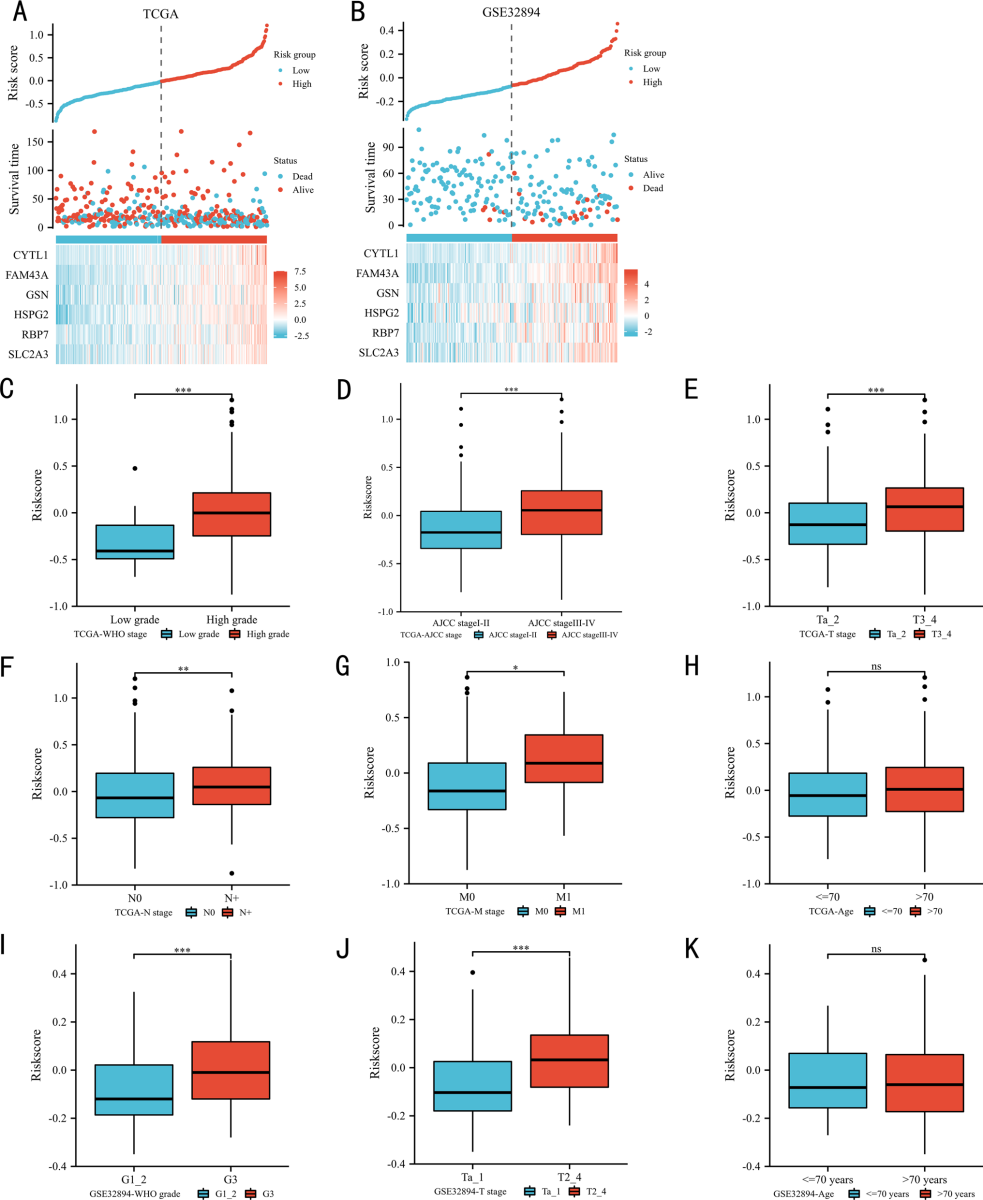
The risk score plots of TCGA (**A**), and GSE32894 (**B**) datasets. The correlation between the index and clinical parameters: TCGA dataset [WHO grade (**C**), AJCC stage (**D**), T stage (**E**), lymph node metastasis stage (**F**), distant metastasis stage (**G**), age (**H**)], GSE32894 dataset [WHO grade (**I**), T stage (**J**), age (**K**)]. N: lymph node metastasis; M: distant metastasis; WHO: World Health Organization; P ≥ 0.05; *, P < 0.05; **, P < 0.01; ***, P < 0.001

There was a significant positive correlation between EPI score and the increase in mortality in the TCGA dataset (Fig. 3A), and GSE32894 dataset (Fig. 3B). In term of TCGA clinical parameters, the EPI score was highly expressed in WHO high grade (Fig. 3C), AJCC III-IV stage (Fig. 3D), T3_4 stage (Fig. 3E), lymph node metastasis (Fig. 3F), and distant metastasis (Fig. 3G). Fortunately, there was no significant difference in age, which might indicate that the EPI score could be applied to people of all age (Fig. 3H). Similarly, in GSE32894 dataset, the EPI score was highly expressed in WHO G3 (Fig. 3I), and T2_4 stage (Fig. 3J). Of course, there was also no significant difference in age (Fig. 3K).

**Fig. 4:**
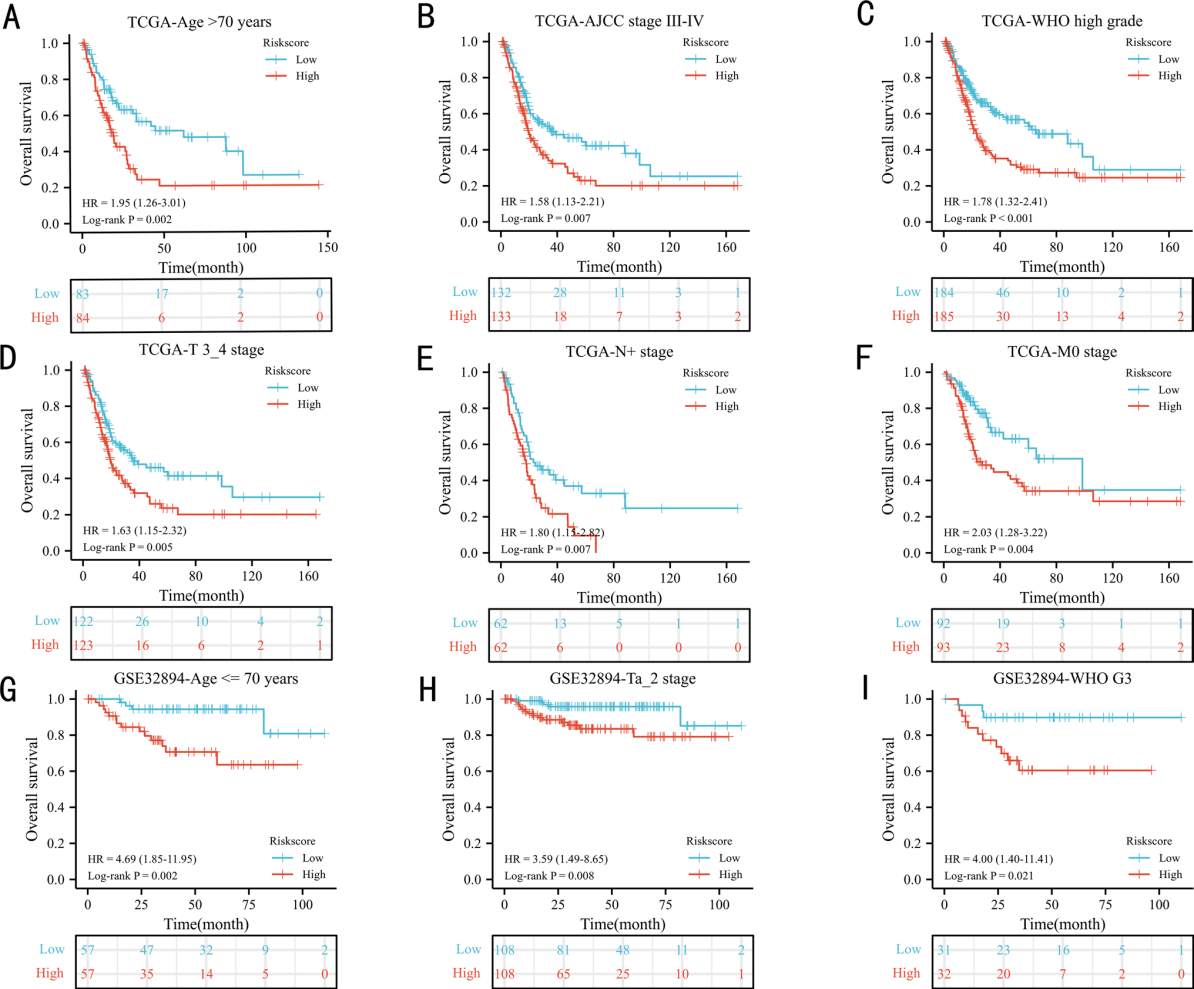
The prognostic ability of the index in clinical subgroups: Kaplan‒Meier analysis results of subgroups in TCGA dataset [age > 70 years (**A**), AJCC stage III-IV (**B**), WHO high grade (**C**), T3_4 stage (**D**), lymph node metastasis (**E**), and no distant metastasis (**F**)], GSE32894 dataset [age <  = 70 years (**G**), T a_2 stage (**H**), and WHO G3 (**I**)]. N: lymph node metastasis; M: distant metastasis; WHO: World Health Organization

The next section of the study was concerned with the prognostic value of EPI in clinical subgroups. The EPI could significantly predict the OS of TCGA patients in many clinical subgroups, such as age > 70 years (Fig. 4A, P = 0.002), AJCC III-IV stage (Fig. 4B, P = 0.007), WHO high grade (Fig. 4C, P < 0.001), T3_4 stage (Fig. 4D, P = 0.005), lymph node metastasis (Fig. 4E, P = 0.007), no distant metastasis (Fig. 4F, P = 0.004) subgroups. In GSE32894 dataset, consistent with the results of TCGA, patients with high EPI score had statistically worse OS than those with low EPI score in age ≤ 70 years (Fig. 4G, P = 0.002), Ta_2 stage (Fig. 4H, P = 0.008), and WHO G3 (Fig. 4I, P = 0.021) subgroups.

**Fig. 5:**
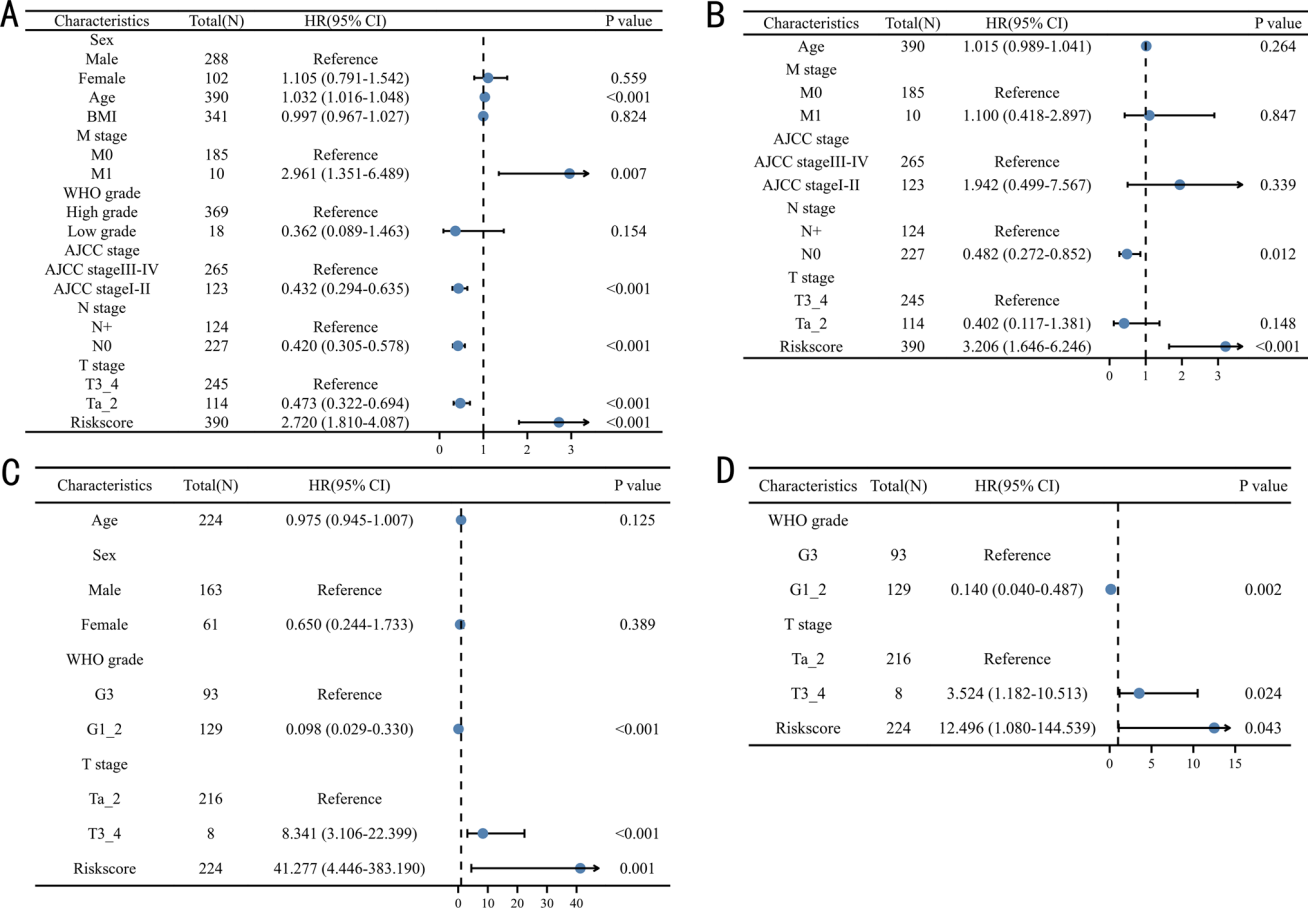
Validation of the independent prognostic value of the cluster: univariable (**A**) and multivariable (**B**) Cox regression model in TCGA dataset, univariable (**C**) and multivariable (**D**) Cox regression model in GSE32984 dataset, N: lymph node metastasis; M: distant metastasis; WHO: World Health Organization

To assess the independent prognostic value, univariable and multivariable Cox regression analysis were used to analyze the prognostic value of EPI in TCGA, and GSE32894 datasets. In TCGA dataset, a multivariable COX model consisting of age, distant metastasis stage, AJCC stage, lymph node metastasis stage, T stage, and EPI (Fig. 5A) indicated that EPI could independently predict the prognosis of BC patients (Fig. 5B, P < 0.001). Similarly, in GSE32894 dataset, a multivariable COX model consisting of WHO grade, T stage, and EPI (Fig. 5C) demonstrated that EPI had independent prognostic value for BC patients (Fig. 5D, P = 0.043).

### The results of function analysis

**Fig. 6:**
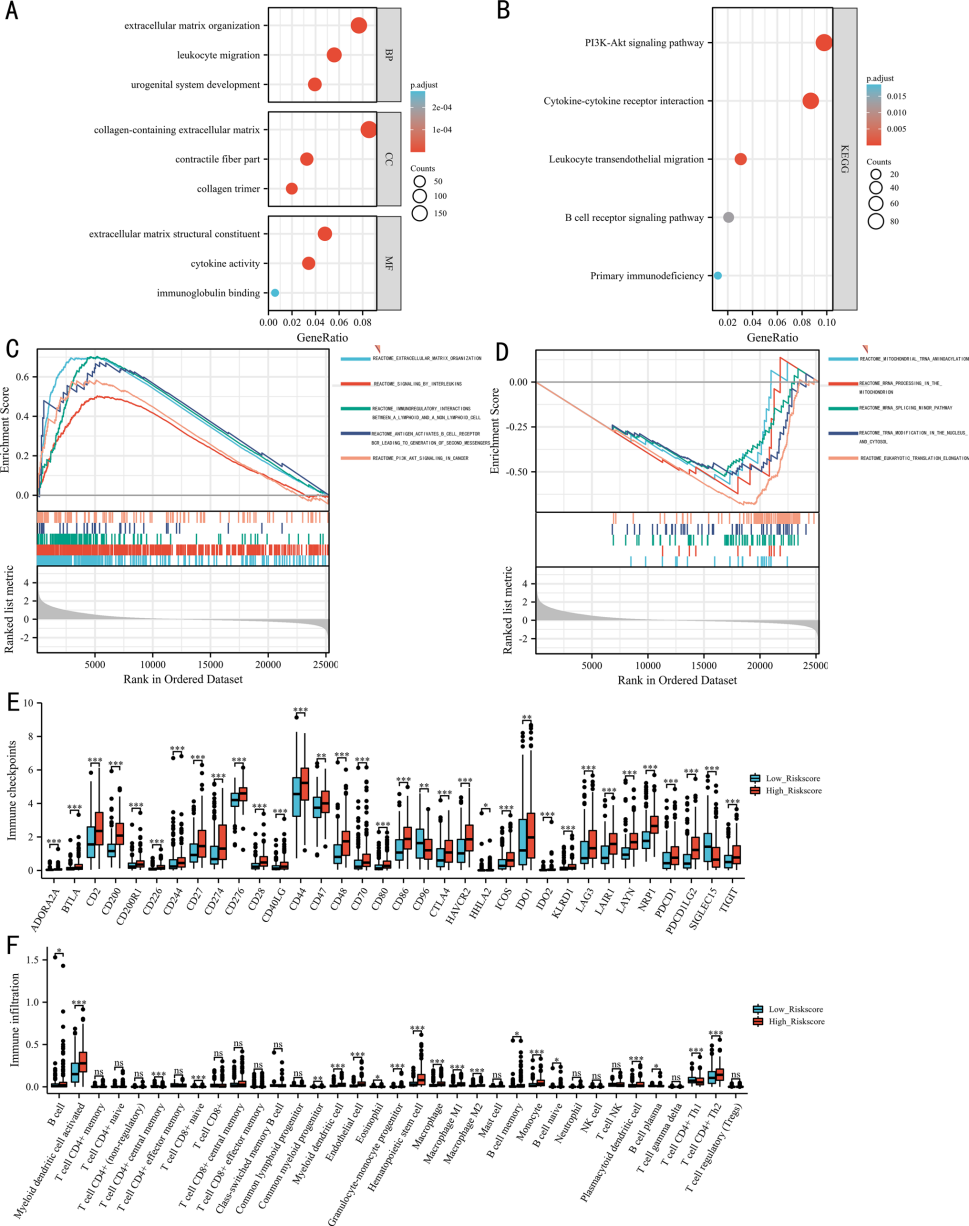
Function analysis: the Gene Ontology results (**A**), Kyoto Encyclopedia of Genes and Genomes results (**B**), Gene Set Enrichment Analysis results (**C**, **D**). Immune checkpoints (**E**) and immune infiltration (**F**). P ≥ 0.05; *, P < 0.05; **, P < 0.01; ***, P < 0.001

Based on TCGA data, GO enriched extracellular matrix related results and immune-related results, such as extracellular matrix organization, collagen-containing extracellular matrix, cytokine activity, leukocyte migration, immunoglobulin binding, and so on (Fig. 6A). Similarly, KEGG enriched immune-related pathways, including cytokine-cytokine receptor interaction, leukocyte trans-endothelial migration.

B cell receptor signaling pathway, and so on (Fig. 6B). Consistent with the results of GO and KEGG analysis, GSEA also enriched extracellular matrix related pathways and immune-related pathways, such as extracellular matrix organization, signaling by interleukins, immunoregulatory interactions between a lymphoid and a non-lymphoid cell, antigen activates B cell receptor BCR leading to generation of second messengers, and so on (Fig. 6C). Furthermore, the results of GSEA indicated that EPI was involved in the regulation of various RNAs, including mitochondrial tRNA aminoacylation, rRNA processing in the mitochondrion, mRNA splicing minor pathway, tRNA modification in the nucleus and cytosol (Fig. 6D).

### The results of immune-related analysis

Since the function results indicated that EPI was involved in the regulation of immune-relate pathways, we explored the role of EPI in the immune checkpoints, tumor immune microenvironment, TMB, stemness index, and immunotherapy. As shown in Fig. 6E, samples in the high-risk score group were positively associated with the expression of CD274, CD47, CTLA4, LAG3, PDCD1, PDCD1LG2, and so on. CD96 and SIGLEC15 were highly expressed in the low-risk score group. Based on the results of xCELL, samples in the high-risk score group were positively correlated with B cell, activated myeloid dendritic cell, common myeloid progenitor, myeloid dendritic cell, eosinophil cell, granulocyte-monocyte progenitor, hematopoietic stem cell, macrophage M1, macrophage M2, B memory cell, monocyte cell, B naïve cell, plasmacytoid dendritic cell, B plasma cell, and CD4+ Th2 cell infiltration (Fig. 6F). CD4+ T central memory cell, CD8+ T naïve cell, and CD4+ Th1 cell were highly infiltrated in the low-risk score group.

**Fig. 7:**
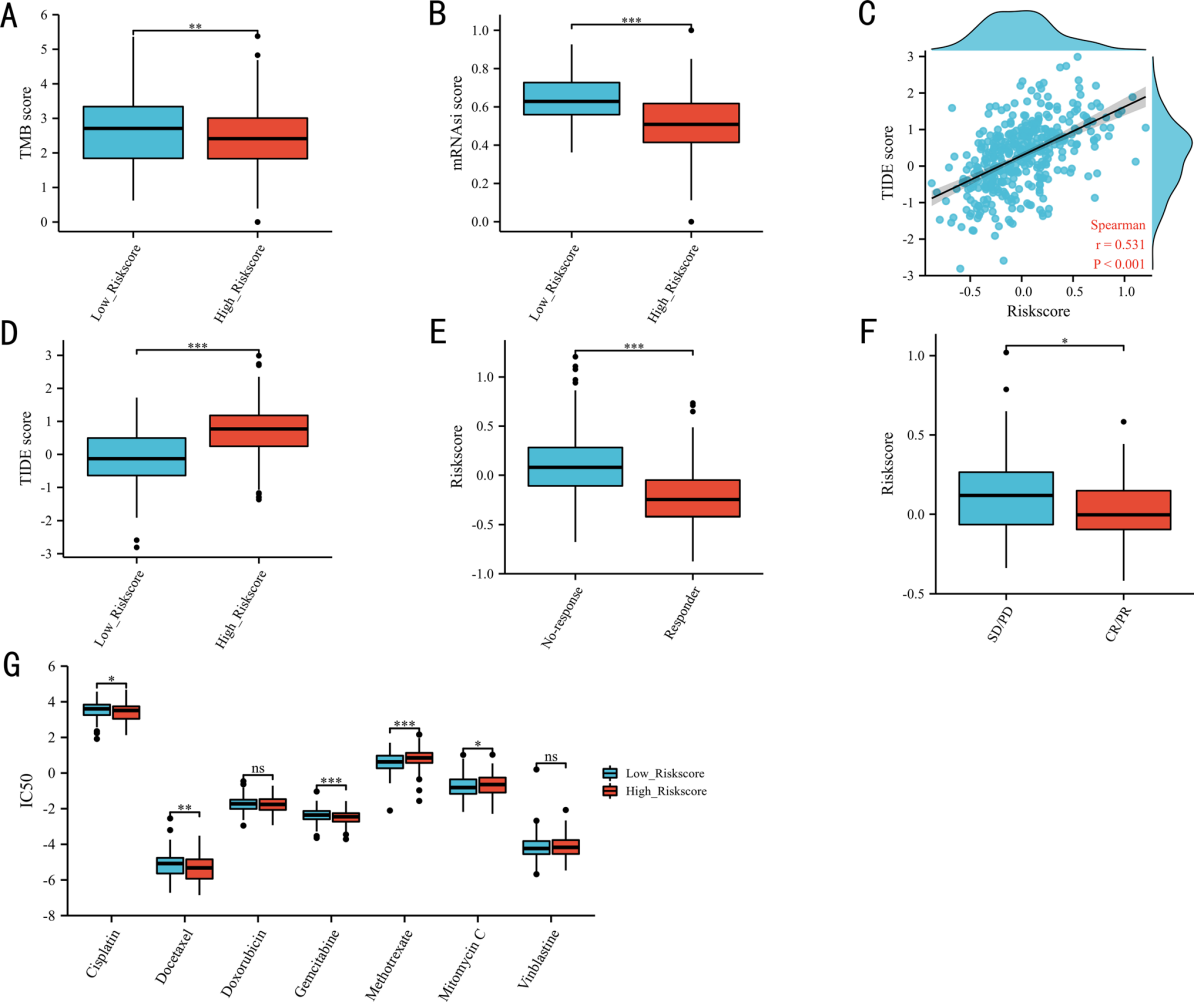
Immune-related analysis based on TCGA dataset: the tumor mutational burden between the high- and low-risk score groups (**A**), the stemness index between the high- and low-risk score groups (**B**), the correlation between the TIDE score and the risk score (**C**), the TIDE score between the high- and low-risk score groups (**D**), the risk score between the response and resistance groups in TIDE (**E**), the risk score between the response and resistance groups in IMvigor210 (**F**). Chemosensitivity in the high- and low-risk score groups (**G**). TMB: tumor mutational burden; mRNAsi: stemness index; IC50: the half-maximal inhibitory concentration, CR: complete response, PR: partial response, SD: stable disease, PD: progressive disease. P ≥ 0.05; *, P < 0.05; **, P < 0.01; ***, P < 0.001

Patients in low-risk score group had significantly higher TMB score (Fig. 7A) and mRNAsi score (Fig. 7B), which might suggest that patients with low-risk score were more to benefit from the immunotherapy. From the Fig. 7C we could see that a positive association was identified between TIDE score and EPI. Of course, patients in high-risk score had statistically higher TIDE score than those in low-risk score group (Fig. 7D). Meanwhile, immunotherapy responders had significantly lower risk score that those with immunotherapy resistance (Fig. 7E). According to the results of data acquiring from real world, responders who accepted anti-PD-L1 medicine had significantly lower-risk score than patients without response (Fig. 7F), which further identified that patients with low-risk score were more to benefit from the immunotherapy. Due to endothelial cells can induce chemotherapy resistant, we also explored the correlation between EPI and chemotherapy. The results showed that the IC50 of cisplatin, docetaxel and gemcitabine was higher in the low-risk score group, while the IC50 of methotrexate and mitomycin was higher in the high-risk score group (Fig. 7G).

## Discussion

As an important element of tumor microenvironment, endothelial cells can communicate with BC cells [34]. For instance, BC cells can secrete soluble ephrin A1 to regulate its receptor EPHA2 on endothelial cells, leading to endothelial cell activation and promoting angiogenesis [35]. Meanwhile, BC cells can also recruit endothelial cells to promote tumor migration and invasion through stimulating CXCL signaling [36]. Thus, we explored the function of endothelial-related genes in BC in this study. We successfully constructed and validated an endothelial-related prognostic index and identified that it could predict the response to immunotherapy or chemotherapy in BC patients.

Locating on human chromosome 4p15-p16, Cytokine-like protein 1 (CYTL1) is a protein coding gene [37]. CYTL1 can induce sprouting and vessel formation by activating endothelial cells [38]. Family with sequence similarity 43 member A (FAM43A) can predict the prognosis of triple-negative breast cancer [39]. Gelsolin (GSN) can predict the prognosis of BC patients [40]. Furthermore, the upregulation of GSN can suppress the metastasis of BC [41]. Heparan Sulfate Proteoglycansulfate proteoglycan 2 (HSPG2) can interact with VEGFR2 at the surface of endothelial cells and has an anti-angiogenesis effect [42, 43]. Retinol Binding Protein retinol binding protein 7 (RBP7), a member of the cellular retinol-binding protein (CRBP) family, can independently predict the prognosis of colon cancer patients and promote the migration and invasion of colon cancer cells [44]. Solute Carrier Family 2 Member 3 (SLC2A3) is a protein coding gene and can predict the OS of BC patients [45]. ASPSCR1 gene, the interacting gene of EPI, can translocate with TFE3 gene and results in a ASPSCR1-TFE3 fusion protein, which causes the tumor hypoxia and angiogenesis in alveolar soft part sarcoma [46]. Moreover, patients with ASPSCR1-TFE3 fusion renal cell cancer may benefit from antiangiogenic based treatment [47]. Therefore, we believed that the EPI was truly associated with endothelial cells in tumor microenvironment.

Given the clinical application of EPI, we set strict inclusive criteria and a total of six endothelial-related genes were selected out finally. According to the introduction of these genes, they were correlated with the function of endothelial cells in tumor micro environment and the development of BC. EPI had prognostic value in internal and external validation datasets, which might suggest that EPI had stable prognostic value for BC patients. Furthermore, for further identification the independent prognostic value of EPI, we employed multivariable COX model to assess EPI and the results demonstrated that EPI can independently predict the prognosis of BC patients in internal and external validation datasets. Furthermore, EPI was significantly associated with poor clinical parameters, such as WHO high grade, T stage, and so on. Meanwhile, there was no difference between EPI and age and sex, which mean that the EPI could predict the prognosis of BC regardless of age and sex. Compared with nonmalignant tissues, pericyte coverage around tumor endothelial cells was lower, which would lead to hypoxia and hypoxia-induced nutrient deprivation [18, 48]. Pericyte coverage can predict the progression-free survival of non-muscle invasive BC [49]. In addition to the surrounding structure, endothelial cells can also secrete von Willebrand Factor to induce platelet aggregation and blood vessel occlusions, which causes the poor prognosis of BC patients [49]. Moreover, the hypoxia and nutrient deprivation induced by endothelial cells can promote the enrichment of cancer stem cells and resistant to chemotherapy [22, 50]. These results supported that endothelial cells in BC microenvironment could significantly regulate the development of BC and predict the prognosis of BC patients. Meanwhile, they also explained why EPI had significantly clinical value in BC patients.

The results of function analysis indicated that EPI was significantly associated with immune-related activity. Thus, we explored the role of EPI in many immune-related analyses. In immune checkpoints analysis, there were significantly differences between the high- and low-risk score groups, such as CD274, CTLA4, and PDCD1. Immune cells are influenced by various factors and play a key role in immunotherapy [51, 52]. The prediction value of these checkpoints for immunotherapy is unclear now [53]. Therefore, the results could only remain us that the response of immunotherapy was different in the high- and low-risk score groups. In terms of immune infiltration, the low-risk score group had highly CD4+ T central memory cell, CD8+ T naïve cell, and CD4+ Th1 cell infiltration. T memory cell in tumor microenvironment could enhance the efficiency of anti–PD-1 cancer immunotherapy [54]. High T naïve cell infiltration was positively correlated with OS in in metastatic castration-resistant prostate cancer, which was consistent with our survival results [55]. Meanwhile, T naïve cell can differentiate into effector cells which may promote the response of immunotherapy [56]. Many factors could affect the anti-tumor function of CD8+ T cell [57]. Of these, CD4+ Th1 cell can promote the effective antitumor-immunity and induce more durable immune-mediated tumor control than CD8+ T cell [58]. In high-risk group, macrophage cells (macrophage M1 cell, macrophage M2 cell) are significantly associated with poor prognosis in tumors and plays a role in immunosuppression [59]. These findings suggest that patients in low-risk score are more likely to benefit from immunotherapy. For identification the hypothesis, we compared the TMB score between the high- and low-risk score groups. With many studies reported, TMB score was positively correlated with the response of immunotherapy. The mean TMB score of low risk-score group was statistically higher than high-risk score group. Furthermore, the cancer stem cells in tumor microenvironment would be enriched by the hypoxia and hypoxia-induced nutrient deprivation. Consistent with the results of TMB score, the low-risk score group had significantly higher mRNAsi score than the high-risk score group. Patients with high mRNAsi score have higher response of immunotherapy than those with low mRNAsi score [30]. Combination of the results of TMB and mRNAsi analysis, we believe that patients in low-risk score are more likely to benefit from immunotherapy. Then, TIDE algorithm was employed to assess the possible response of immunotherapy in each included TCGA patient. After identifying the positive correlation between TIDE score and the risk score, the low-risk score group had significantly lower TIDE score than the high-risk score group. High TIDE score was usually correlated with immunotherapy resistance [31]. Meanwhile, compared immunotherapy resistant patients, immunotherapy responders had statistically lower risk score. In the results of data from real world, immunotherapy responders had significantly lower risk score than immunotherapy resistant patients. The above results support that patients in low-risk score are more likely to benefit from immunotherapy.

Chemosensitivity also evaluated in the low- and high-risk score group due to endothelial cells could induce chemotherapy resistance [22, 23]. Interesting, while the high-risk score group might not suitable for immunotherapy, the high-risk score group was sensitive to cisplatin, docetaxel, and gemcitabine. Given the poor prognosis of patients in the high-risk score group, we suggest them should accept chemotherapy as soon as possible. With the advent of the aging era, the economic and mental burden of BC is increasingly heavy. Precision medicine can effectively alleviate or even solve this problem at a lower cost. In this study, we successfully constructed and validated an endothelial-related prognostic index. Moreover, we found patients in high- and low-risk core groups were sensitive to different treatments. These results based on the integrating single-cell and bulk RNA sequencing data from bladder cancer patients, which are more reliable and have strong clinical relevance.

Some limitations should be mentioned in this research. Our data, sourced from the TCGA and GEO databases (specifically GSE32894), involved a limited sample size, potentially leading to result deviations. A deeper investigation into key genes is required. Future experiments are needed to explore the molecular biological functions of six key genes.

## Conclusion

By integrating single-cell and bulk RNA sequencing data from bladder cancer patients, we successfully identified the key genes from the perspective of endothelial cells in tumor microenvironment and constructed an endothelial-related prognostic index which had positive implications in precision medicine.

### Supplementary Information

Additional file1 (XLSX 17 KB)

Additional file2 (XLSX 34 KB)

Additional file3 (DOCX 16 KB)

## Data Availability

All data from this study were downloaded from online databases. Therefore, everyone can get the data online. Further inquiries can be directed to the corresponding author.
