# Supplementary material for: An endothelial-related prognostic index for bladder cancer patients
Source: Discov Oncol. 2024 Apr 25;15:128. doi: 10.1007/s12672-024-00992-4 (PMC11045713; doi:10.1007/s12672-024-00992-4)
Supplement: Supplementary file 3 — Additional file3 (DOCX 16 KB) [file 12672_2024_992_MOESM3_ESM.docx]

Supplementary table 3. The clinicopathological characteristics of the GSE32894 included patients.

| Characteristic | Low risk-score | High risk-score | p |
| --- | --- | --- | --- |
| n | 112 | 112 |  |
| Age, mean ± SD | 68.75 ± 12.24 | 70.12 ± 10.24 | 0.366 |
| Sex, n (%) |  |  | 0.368 |
| Female | 27 (12.1%) | 34 (15.2%) |  |
| Male | 85 (37.9%) | 78 (34.8%) |  |
| WHO grade, n (%) |  |  | < 0.001 |
| G1_2 | 80 (36%) | 49 (22.1%) |  |
| G3 | 32 (14.4%) | 61 (27.5%) |  |
| T stage, n (%) |  |  | 0.722 |
| T3_4 | 3 (1.3%) | 5 (2.2%) |  |
| Ta_2 | 109 (48.7%) | 107 (47.8%) |  |
| Overall survival, n (%) |  |  | 0.011 |
| Alive | 106 (47.3%) | 93 (41.5%) |  |
| Dead | 6 (2.7%) | 19 (8.5%) |  |

SD: Standard deviation; WHO: World Health Organization; n: Number.
